# ZCCHC17: a target for synaptic dysfunction and neuronal excitability in Alzheimer’s disease

**DOI:** 10.3389/fnagi.2026.1737060

**Published:** 2026-02-10

**Authors:** Brittany A. Klub, Andrew F. Teich, Giuseppe P. Cortese

**Affiliations:** 1 Touro College of Osteopathic Medicine, Touro University, Great Falls, MT, United States; 2 Department of Pathology and Cell Biology, Columbia University Irving Medical Center, New York, NY, United States; 3 Taub Institute for Research on Alzheimer’s Disease and the Aging Brain, Columbia University Irving Medical Center, New York, NY, United States; 4 Department of Neurology, Columbia University Irving Medical Center, New York, NY, United States; 5 McLaughlin Research Institute, Great Falls, MT, United States

**Keywords:** Alzheimer’s disease (AD), neuronal excitability, neuronal hyperexcitability, synaptic dysfunction, synaptic gene expression, ZCCHC17

## Abstract

Epileptic activity and neuronal excitability have been reported in the setting of Alzheimer’s disease (AD), and may be linked to disease progression and severity. A shift in the excitation/inhibition balance to favor a more excitatory-dominant outcome appears to underlie the overall hyperactivity, with key mechanisms known to regulate excitatory and inhibitory neurotransmission in the brain being primarily affected. Synaptic dysfunction is a critical event in AD pathogenesis. Recent research suggests that the zinc finger protein, ZCCHC17 (Zinc Finger CCHC-Type Containing 17), serves as a potential master regulator of synaptic dysfunction in AD, with expression significantly reduced in the AD brain prior to gliosis and neuronal loss. Reduced levels of ZCCHC17 have been shown to lead to abnormal RNA processing and neuronal hyperexcitability. This review examines the specific role of ZCCHC17 in the AD brain, and discusses how ZCCHC17 may regulate mechanisms that underlie neuronal hyperexcitability. New insight into synaptic regulators of disease may contribute to improvements in early-stage diagnostics and interventions, and may better guide therapeutic approaches aimed at rescuing synaptic dysfunction in the prodromal stages of AD.

## Introduction

Alzheimer’s disease (AD) is the most common cause of dementia (Alzheimer’s and Dementia, 2024). The accumulation of extracellular amyloid plaques composed of amyloid beta-protein (Aβ) and the formation of intracellular neurofibrillary tangles (NFTs) of hyperphosphorylated tau are considered key pathological hallmarks of AD (Glenner and Wong, 1984; Grundke-Iqbal et al., 1986), and have therefore been a target for research and guided therapeutic approaches and diagnostic criteria. However, over the past few decades, AD treatments targeting these protein aggregates have yielded modest outcomes at best, resulting in high failure rates and limited effects among drug candidates (Klyucherev et al., 2022). There is strong evidence confirming that plaque formation and NFTs are associated with neurodegeneration (Chinnathambi et al., 2025). However, the role of plaques and NFTs as early molecular mechanisms remains in question, suggesting that additional factors surrounding AD pathogenesis and pathophysiology may also play a contributing role.

Significant efforts have been directed at uncovering interactions between protein aggregates and physiological and immunological irregularities, which include neuroinflammation, mitochondrial dysfunction, oxidative stress, and epileptiform activity (Cortese et al., 2024; Csernus et al., 2022; Gourmaud et al., 2022; Hering et al., 2025; Klyucherev et al., 2022; Tönnies and Trushina, 2017; Vossel et al., 2017). Studying these physiological and immunological processes may be useful for understanding early prodromal events prior to the onset of symptoms, and may also inform our understanding of disease progression and severity.

Neuronal hyperactivity is detected in the early stages of AD (Barbour et al., 2024; Cretin et al., 2016; Vossel et al., 2013, 2016), and may accelerate disease progression and cognitive decline (Baker et al., 2019; Horvath et al., 2021; Lott et al., 2012; Volicer et al., 1995; Vossel et al., 2017). Similar neurophysiological changes have been found in AD animal models (Barbour et al., 2024; Busche et al., 2008; Ciccone et al., 2019; Maier et al., 2014; Minkeviciene et al., 2009; Nygaard et al., 2015; Palop et al., 2007; Palop and Mucke, 2009, 2010; Sanchez et al., 2012; Zou et al., 2024). Aβ and hyperphosphorylated tau have been linked to neural hyperactivity in AD (Lam et al., 2022; Palop and Mucke, 2009; Vicente et al., 2024), and also associated with seizure disorders (Hwang et al., 2022; Romoli et al., 2021; Thom et al., 2011; Vicente et al., 2024). Mechanistically, AD-associated hyperactivity is thought to occur following disruptions to pre-, post-, and peri-synaptic mechanisms that underlie excitatory and inhibitory neurotransmission, shifting the balance of excitatory/inhibitory (E/I) activity in the brain (Barbour et al., 2024; Lauterborn et al., 2021). Numerous studies have confirmed that aberrant glutamatergic and GABAergic signaling disrupts the normal balance of E/I activity (Gong et al., 2009; Lauterborn et al., 2021; Limon et al., 2012; Palop et al., 2007; Vossel et al., 2017; Wakabayashi et al., 1999). Thus, identifying mechanisms, or key players contributing to the shift in E/I activity may be critical for understanding early molecular events responsible for AD pathogenesis. In addition, it is possible that comorbid conditions, including epileptic activity, may not only be correlated with AD but may also contribute significantly to AD pathogenesis.

This review discusses recent findings that characterized a novel synaptic mechanism by which synaptic dysfunction and hyperexcitability occurs in AD. Specifically, we highlight the initial discovery that ZCCHC17, a master regulator of synaptic gene expression (Bartosch et al., 2024; Tomljanovic et al., 2018), which is significantly reduced in AD brain prior to gliosis and neuronal loss (Tomljanovic et al., 2018) and causes neuronal hyperexcitability in a neuronal model (Cortese et al., 2024). This provides a potential novel target for diagnostic and therapeutic strategies to preserve cognitive function surrounding AD pathology.

## Synaptic dysfunction in AD

It is widely recognized that synaptic failure is an early event in AD (Selkoe, 2002), and is thought to underlie cognitive impairment during the earliest clinical phases of the disease (Chen et al., 2019; Li and Selkoe, 2020). Synapse loss precedes overall neuronal loss and correlates with premortem cognitive status (de Wilde et al., 2016; DeKosky and Scheff, 1990; Hamos et al., 1989; Robinson et al., 2014; Terry et al., 1991). Aβ and tau may have normal roles at the synapse that is relevant for how these protein aggregates contribute to neurodegeneration, which directly links synaptic dysfunction to the two disease-defining proteins of Alzheimer’s disease (Spires-Jones and Hyman, 2014). Additional evidence linking Alzheimer’s disease to neuronal hyperactivity comes from studying genetically influenced disease. For example, homozygous carriers of the ε4 allele of Apolipoprotein E (APOE4), a well-established genetic risk factor AD, show an increased risk for late-onset epilepsy with dementia (Johnson et al., 2018; Liang et al., 2019). Furthermore, patients with familial forms of AD have a seizure rate approaching 30% in some studies (Shea et al., 2016). Similarly, individuals with Trisomy 21 (Down syndrome) have an increased risk of seizures (Rahman and Fatema, 2019). Despite all this evidence, the specific mechanisms by which genetic risk promotes hyperexcitability, and the chain of causality, are not well understood.

## Epileptiform activity in AD

Similarly to genetic risk studies, it has also been noted that AD patients in the general population have an elevated risk for developing seizures and epilepsy (Horváth et al., 2016, 2018; Vossel et al., 2013). More than 40% of AD patients present with a subclinical epileptiform activity (SEA), as characterized by isolated epileptiform discharges without overt epileptic seizures (Horváth et al., 2018; Horvath et al., 2021; Vossel et al., 2013, 2016). Reports of overt seizure in AD range widely in the literature, although most studies show increased risk, ranging up to 20% of patients over the disease course in some studies (Yang et al., 2022), with common occurrences in younger AD patients (Sherzai et al., 2014; Vossel et al., 2013). Periods of network hyperexcitability and SEA within the brain are known to occur during the early, presymptomatic stages of AD (Cretin et al., 2016; Quiroz et al., 2010; Ranasinghe et al., 2022; Sarkis et al., 2016; Sepulveda-Falla et al., 2012; Vossel et al., 2013, 2016). It’s worth noting, however, that similar activity has been shown to occur during later stages of AD (Hauser et al., 1986; Sherzai et al., 2014; Vossel et al., 2017). Given the difficulty in observing non-motor seizures in patients, most SEA and hyperactive events go undetected. Several studies in AD patients and animal models have confirmed the pathogenicity surrounding these hyperactive events (Baker et al., 2019; Barbour et al., 2024; Hector and Brouillette, 2020; Horváth et al., 2016, 2018; Vossel et al., 2013, 2017). Given that the balance between excitatory and inhibitory neurotransmission, as determined by postsynaptic currents through excitatory glutamate and inhibitory GABA signaling, is necessary for normal network function (Barral and D Reyes, 2016; Zhou and Yu, 2018), it has been proposed that shifting of the E/I balance to favor excitatory glutamatergic neurotransmission may contribute to the overall network hyperactivity in AD (Lauterborn et al., 2021; Vicente et al., 2024). Furthermore, Lauterborn et al. (2021) confirmed that the E/I imbalance favoring hyperexcitability in AD can occur despite synapse loss. Excess glutamate activity and reduced GABAergic synaptic activity occur in AD and epilepsy, potentially leading to excitotoxicity and driving the neurodegeneration seen later in AD pathology (Barker-Haliski and White, 2015; Calvo-Rodriguez and Bacskai, 2021; Vicente et al., 2024; Yu et al., 2025). Presynaptic (Akyuz et al., 2021; Anschuetz et al., 2024; Fukata and Fukata, 2017; Jiang et al., 2025; Sze et al., 1997), perisynaptic (Akyuz et al., 2021; Anschuetz et al., 2024; Fukata and Fukata, 2017; Jiang et al., 2025; Sze et al., 1997), and postsynaptic (Akyuz et al., 2021; Brines et al., 1997; Escamilla et al., 2024; Fukata and Fukata, 2017; Govindpani et al., 2020; Kwakowsky et al., 2018; Mathern et al., 1997, 1998; Ning et al., 2024; Osse et al., 2023) changes are commonly observed.

## ZCCHC17 and AD

Gueydan et al. (2002) first discovered ZCCHC17 (Zinc Finger CCHC-Type Containing 17) while screening a cDNA library for RNA binding proteins. Additionally, ZCCHC17 (also known as pNO40) was independently identified by Chang et al. (2003) via a yeast 2-hybrid screen for pinin-interacting proteins. ZCCHC17 has an S1 RNA-binding domain and a zinc-finger (CCHC) domain, with two nuclear localization signals. Although highly expressed in brain, ZCCHC17 transcripts are found throughout the body, including in heart, skeletal muscle, and thymus (Chang et al., 2003). Current evidence suggests that ZCCHC17 has roles in both mRNA (Lin et al., 2017) and rRNA (Lin et al., 2019) processing, and that it may coordinate a variety of homeostatic cellular functions (Lin et al., 2017).

**FIGURE 1:**
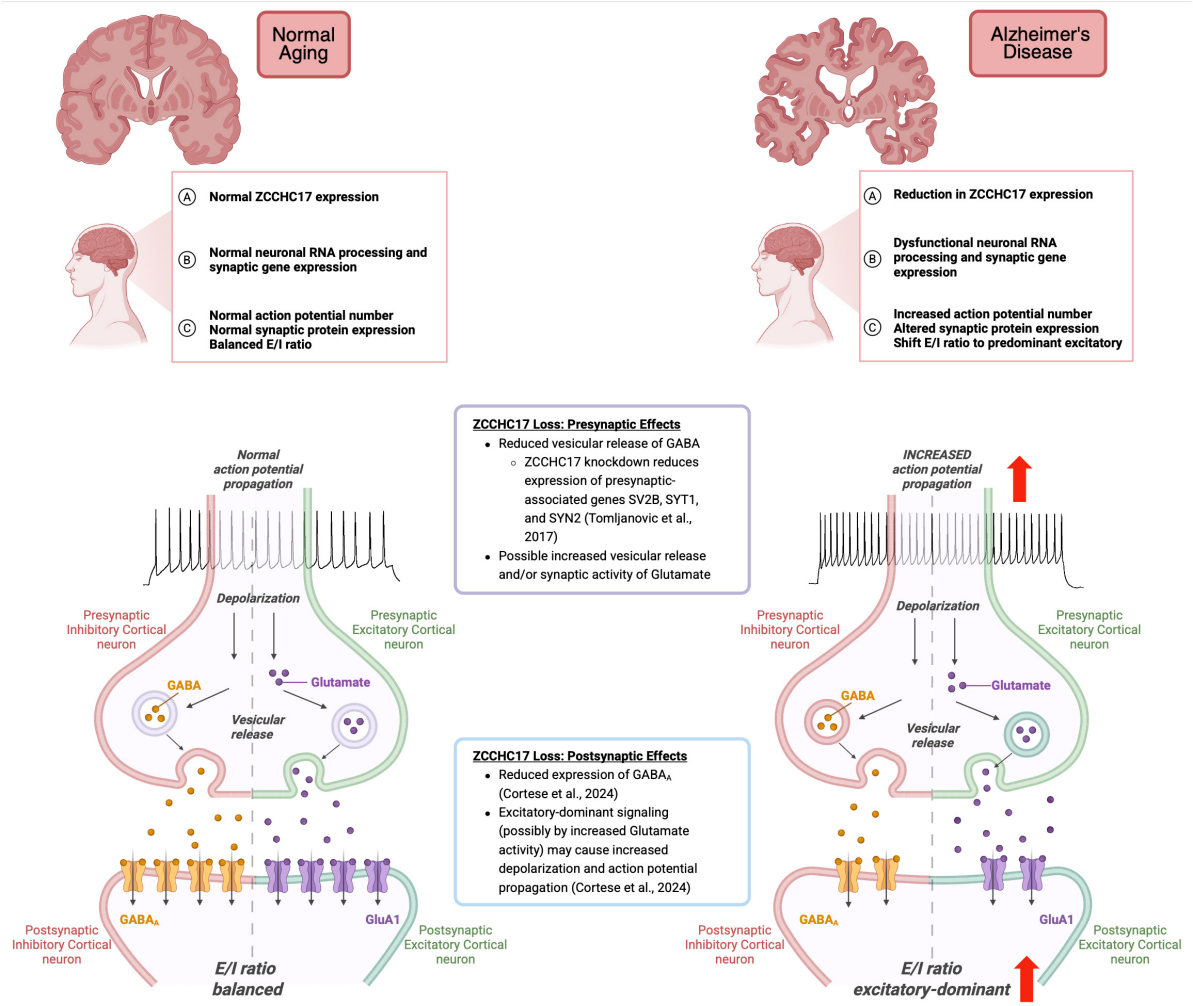
The physiological and biological consequences of reduced ZCCHC17 in the AD brain. Created in BioRender. Cortese, G. (2026) https://BioRender.com/d61fup3.

More than a decade after the discovery of ZCCHC17, ZCCHC17 was shown to be implicated in AD pathology (Li et al., 2015; Tomljanovic et al., 2018). Using novel data mining techniques to identify molecular drivers of synaptic dysfunction in AD, Tomoljanovic et al. (2018) demonstrated that ZCCHC17 is normally expressed in neurons and is reduced in expression in human AD tissue from temporal cortex during the early course of pathology prior to significant gliosis and neuronal loss. Subsequently, they modeled ZCCHC17 knockdown in primary cortical neurons, and confirmed that loss of ZCCHC17 leads to reduced expression of several dozen synaptic targets; including presynaptic genes SV2B (Synaptic Vesicle Glycoprotein 2B), SYT1 (synaptotagmin-1), and SYN2 (synapsin 2), suggesting a role as a transcriptional regulator whose dysfunction in AD contributes to synaptic dysregulation (Tomljanovic et al., 2018) (see Figure 1 for summary of discussion in this section). Building on this work, Bartosch et al. (2024) showed that ZCCHC17 knock-down in human iPSC-derived neurons partially reproduces synaptic gene-splicing abnormalities seen in AD brain tissue, and further showed that ZCCHC17 expression correlates with cognitive resilience in the setting of AD pathology. Interestingly, the work of Bartosch et al. (2024) also uncovered an APOE4-dependent correlation of ZCCHC17 expression with NFT burden, and further showed that ZCCHC17 knock-down and tau overexpression lead to shared spicing abnormalities in neurons, suggesting a relationship between tau dysfunction and ZCCHC17 impairment. In an attempt to study the functional consequences of impaired ZCCHC17 function, Cortese et al. (2024) demonstrated that loss of ZCCHC17 partially phenocopies AD-related loss of synaptic proteins and hyperexcitability. Using an *in vitro* model of siRNA knockdown of ZCCHC17 in primary cortical neurons, Cortese et al. (2024) demonstrated that there was a shift in excitatory (glutamatergic) and inhibitory (GABAergic) neurotransmission, favoring an excitatory-dominant system that caused neuronal hyperactivity on a single-cell level, and showed that these changes are accompanied by reduced levels of postsynaptic glutamate (GluA1) and GABA_A_ receptors and postsynaptic scaffold proteins Shank3 and Gephyrin. Note that while Cortese et al. (2024) did not show how reduced excitatory and inhibitory input leads to overall hyperexcitability in their model, others have found similar results in AD tissue and shown that there is an overall net increase in excitation after loss of both excitatory and inhibitory inputs (Lauterborn et al., 2021). It should also be noted that the exact role of ZCCHC17 in the nucleus is still being investigated, and this raises questions as to how ZCCHC17 loss leads to reduced expression and aberrant splicing of synaptic genes. Outstanding questions aside, the above findings have furthered our understanding of the functional role of ZCCHC17 knock-down in neurons, and may provide a new perspective for understanding and targeting early events in AD.

## Conclusion

In conclusion, a multifaceted network of presynaptic, perisynaptic, and postsynaptic dysregulation leads to neuronal hyperexcitability. Recent studies have documented comparable physiological phenotypes in AD brain and have shed further light on the role of synaptic dysfunction in AD and its value as a potential therapeutic target. ZCCHC17 is reduced in the AD brain before gliosis and neuronal loss, and studies have supported a role for ZCCHC17 in AD-related synaptic dysfunction. Specifically, reductions in ZCCHC17 have been shown to result in: (1) decreased expression of several synaptic genes (Tomljanovic et al., 2018), (2) abnormal RNA processing affecting synaptic genes (Bartosch et al., 2024), and (3) phenocopies of AD-related loss of synaptic proteins and hyperexcitability (Cortese et al., 2024). Converging data suggests that mechanisms underlying excitatory (glutamatergic) and inhibitory (GABAergic) neurotransmission, including but not limited to presynaptic transporters and vesicle fusion proteins and postsynaptic inotropic and metabotropic receptors, may contribute to AD pathogenesis. (see Figure 1 proposing potential pre- and postsynaptic mechanisms that may give rise to hyperexcitability). Future studies aimed at determining how impaired ZCCHC17 function impacts synaptic function in AD are merited. Further exploration of ZCCHC17 function in models of genetic predisposition to AD, like Down syndrome, as well as models of multiple forms of epilepsy, may also illuminate the role of ZCCHC17 as an early target. To date, these studies have not been done but hold promise for providing a novel portal for exploration of AD.
